# Immune Checkpoint Inhibitors: A Narrative Review on PD-1/PD-L1 Blockade Mechanism, Efficacy, and Safety Profile in Treating Malignancy

**DOI:** 10.7759/cureus.58138

**Published:** 2024-04-12

**Authors:** Nicolas D Benelli, Ian Brandon, Karina E Hew

**Affiliations:** 1 Internal Medicine, St. George's University School of Medicine, St. George's, GRD; 2 Family Medicine, Baptist Health South Florida, Miami, USA; 3 Gynecologic Oncology, University of Florida College of Medicine - Jacksonville, Jacksonville, USA

**Keywords:** pd-l1, pd-1 inhibitor, immune checkpoint inhibitors, cancer, cancer immunotherapy

## Abstract

Checkpoint inhibitors have been implicated in the treatment of several cancers due to their ability to exploit the immune system's regulatory pathways. This article serves to emphasize the importance of these immunotherapeutic agents and provide further insight into their mechanisms, efficacies, and safety profiles. The main agents in question include programmed cell death protein 1 (PD-1) and programmed death ligand 1 (PD-L1). Several literature sources were found to assess the use of these inhibitors in cancers involving the lung, breast, and skin. Several peer-reviewed systematic reviews and the outcomes of clinical trials are combined within this article to support the use and further investigation of these agents in treating neoplasms. Further research into these forms of therapy underscores the revolutionary advancement of oncological interventions, which is important given the rising incidence of neoplasms within populations.

## Introduction and background

The American Cancer Society reports millions of new cancer cases being diagnosed annually, making neoplasm a leading cause of patient mortality [1]. Cancer carries a complex etiology that involves a plethora of risk factors including metabolic abnormalities, social behaviors such as smoking or drinking, genetic predisposition, and infectious agents. The pathogenesis of cancer involves a series of events resulting in uncontrolled cell growth leading to the formation of a tumor. The progression of cancer involves a tumor metastasizing to different sites and altering the natural chemistry or physiology within the body to avoid immune detection and enhance oncogenesis.

**Figure 1:**
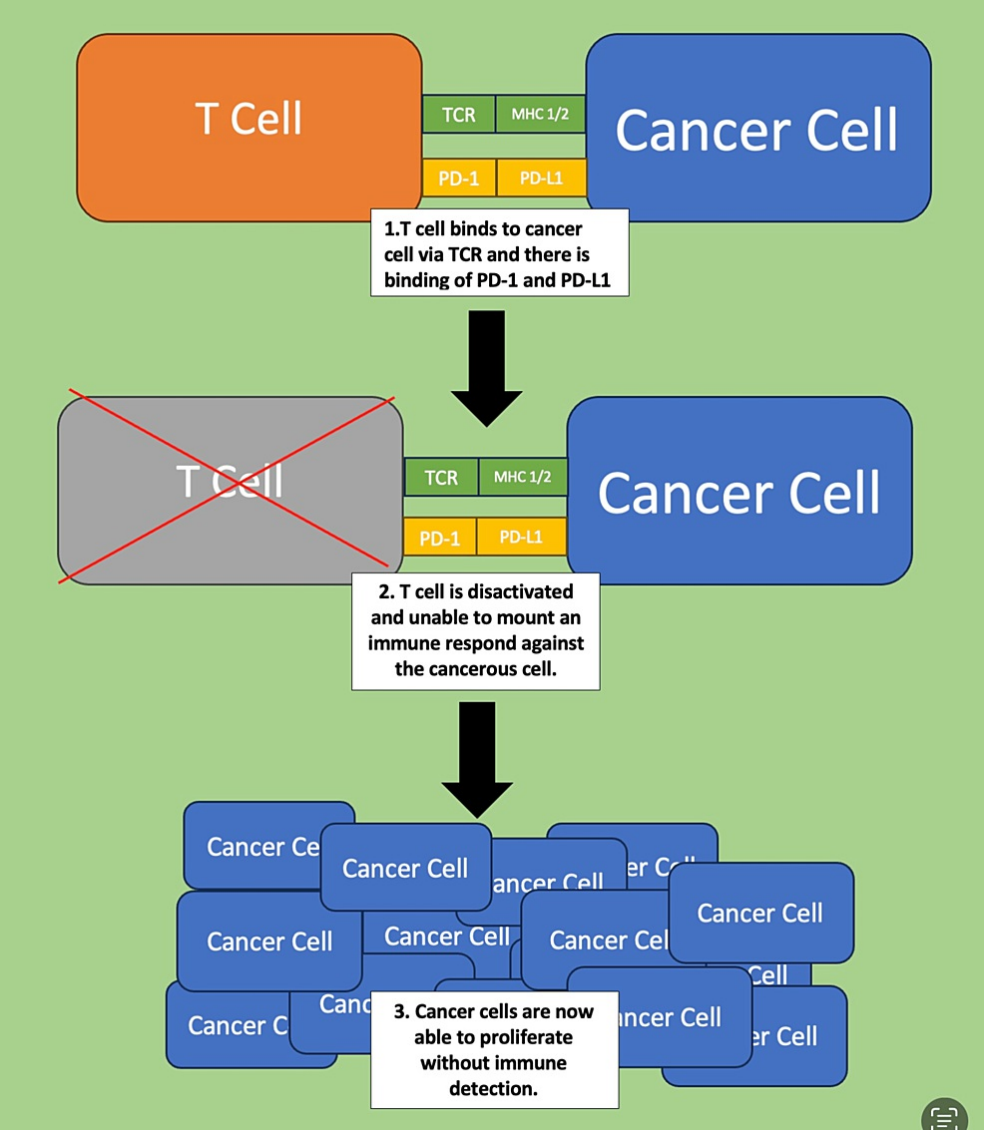
PD-1 and PD-L1 binding Image credit: Nicolas Benelli PD-1: programmed cell death protein 1, PD-L1: programmed death ligand 1, TCR: T-cell receptor, MHC: major histocompatibility complex

One of the ways in which malignant cells may avoid immune detection is by binding to a particular receptor on T-cells: the programmed cell death protein 1 (PD-1) receptor. Programmed death ligand 1 (PD-L1) is a protein that is expressed in various malignant cells. When a tumor cell binds to a T-cell via PD-L1, T-cell activity is suppressed and unable to properly kill the cancer cell [2]. This mechanism can be seen in Figure 1.

Inhibitors of this interaction between T-cells and cancer cells have been developed, and these inhibitors can specifically target PD-1 on T-cells or PD-L1 on tumor cells. These inhibitors may be used as monotherapy or can be combined with traditional chemotherapy to treat a specific cancer. This therapy helps reactivate the immune response to malignant cells and has demonstrated prolonged efficacy in trials involving lung cancer, melanoma, and breast cancer [3]. Some examples of these inhibitors may be seen in Table 1 with their respective targets and which cancers they have shown to have an effective response against.

**Table 1:** Examples of PD-1 and PD-L1 inhibitors PD-1: programmed cell death protein 1, PD-L1: programmed death ligand 1

Agent	Receptor targeted	Cancers that have been treated in clinical trials
Cemiplimab [3]	PD-1	Squamous cell carcinoma, advanced basal cell carcinoma
Nivolumab [3]	PD-1	Melanoma, non-small cell lung cancer, renal cell carcinoma, Hodgkin lymphoma, hepatocellular carcinoma
Pembrolizumab [3]	PD-1	Melanoma, non-small cell lung cancer, squamous cell carcinoma of the head and neck
Durvalumab [3]	PD-L1	Urothelial carcinoma, non-small cell carcinoma
Avelumab [3]	PD-L1	Merkel cell carcinoma, urothelial carcinoma, renal cell carcinoma
Atezolizumab [3]	PD-L1	Bladder cancer, triple-negative breast cancer, non-small cell lung cancer

## Review

Methodology

This review consists of literature that has been peer-reviewed and indexed by multiple platforms including PubMed, Medline, and Embase. Sources included systematic reviews, meta-analyses, clinical trials, and case reports. Key terms that were searched include "immune checkpoint inhibitors," "PD-1 inhibitors," "PD-L1 inhibitors," and "immunotherapy." To ensure that the most recent literature was obtained, filters were incorporated to only include articles and studies after the year 2010.

Efficacy of anti-PD-1/PD-L1 agents

Durvalumab and Its Results Within Clinical Trials

Some of the major clinical trials that investigated the outcomes of using durvalumab for lung cancer include the PACIFIC, MYSTIC, POSEIDON, and NEPTUNE trials. The patients in all trials had unresectable stage 3 non-small cell lung carcinoma (NSCLC). These studies had similar components in terms of sample selection, and the relationship between the results of each study can be of importance in the evaluation of this immunotherapy. The sample selection included patients who were similar in age, smoking status, and staging of cancer. Exclusion criteria included patients with a history of PD-1 inhibitor therapy, autoimmune disease, immunodeficiencies, and grade 2 or higher pneumonitis from previous chemotherapy. A measure of efficacy was assessed by calculating the median progression-free survival (PFS) team after treatment as well as a one-year PFS rate. The results of these three trials are shown in Table 2.

**Table 2:** Results of major clinical trials involving the use of durvalumab for treating NSCLC NSCLC: non-small cell lung carcinoma, PFS: progression-free survival, WD: with durvalumab, ND: no durvalumab

Study	Method	Combination therapy	Objective	Number of patients treated with durvalumab	Number of patients treated without durvalumab	Median PFS (months)	12-month PFS rate	Median age	Current smoking status (%)	Sex (%)
PACIFIC trial (Antonia et al. [4])	Phase III randomized clinical trial	None	Evaluate the effectiveness of durvalumab as consolidation therapy in patients with NSCLC	473	236	WD: 16.8 versus ND: 5.6	WD: 55.9 versus ND: 35.3	WD: 64 versus ND: 64	WD: 16.6 versus ND: 16.6	WD: male: 70.2, female: 29.8; ND: male: 70.0, female: 30.0
POSEIDON trial (Johnson et al. [5])	Phase III randomized clinical trial	Tremelimumab	Evaluate the effectiveness of tremelimumab plus durvalumab versus traditional chemotherapy	335	337	WD: 5.5 versus ND: 4.8	WD: 24.4 versus ND: 13.1	WD: 64.5 versus ND: 64	WD: 18.9 versus ND: 19.6	WD: male: 74.9, female: 25.1
MYSTIC trial (Rizvi et al. [6])	Phase III randomized clinical trial	Tremelimumab	Evaluate whether durvalumab treatment with or without tremelimumab improves survival outcomes in patients with metastatic NSCLC	369	352	WD: 3.9 versus ND: 5.4	WD: 25.8 versus ND: 14.3	WD: 64 versus ND: 64.5	WD: 28.8 versus ND: 24.1	WD: male: 69.3, female: 30.7
NEPTUNE trial (de Castro et al. [7])	Phase III randomized control trial	Tremelimumab	Evaluate first-line durvalumab plus tremelimumab versus chemotherapy in metastatic NSCLC	410	413	WD: 4.2 versus ND: 5.1	WD: 25.6 versus ND: 7.0	WD: 63 versus ND: 65	WD: 33.7 versus ND: 28.3	WD: male: 72.4, female: 27.6

Durvalumab's application in treating NSCLC across four clinical trials reveals compelling evidence of its efficacy and potential implications. When administered to patients (WD), durvalumab demonstrated a marked improvement in PFS and overall survival (OS) rates compared to groups not treated with durvalumab (ND). It is important to note that this improvement in cancer progression was associated with an increased incidence of grade 3/4 adverse events (AEs) such as anemia, pneumonia, and pneumonitis.

The PACIFIC trial emphasized this trend, yielding a median PFS of 16.8 months in the WD group compared to 5.6 months in the ND group. The hazard ratio (HR) for PFS equates to 0.52, indicating that patients receiving durvalumab had a 48% reduction in the risk of disease progression compared to patients who were not receiving this treatment. A critical metric to assess the treatment response was the 12-month PFS rate. This parameter was significantly higher in the WD group compared to the ND group (55.9% versus 35.3%). However, the benefit of durvalumab within this trial was correlated with a higher incidence of grade 3/4 adverse events (AEs) (29.9% in WD versus 26.1% in ND), further emphasizing the importance of continuously monitoring individuals undergoing this immunotherapy. The POSEIDON trial further underlines the efficacy of durvalumab, yielding a median PFS of 5.5 months for the WD group compared to 4.8 months for the ND group. In this trial, the HR for PFS was 0.76, suggesting a 24% reduction in the risk of cancer progression when patients were treated with durvalumab. The 12-month PFS rate for WD was more than double that of ND (24.4% versus 11.2%), highlighting the sustained benefit of durvalumab over time. The efficacy of durvalumab is further supported by the MYSTIC trial, where patients undergoing durvalumab therapy had a median overall survival of 16.3 months compared to the chemotherapy group who yielded a median survival rate of 12.9 months. The group receiving combination therapy with tremelimumab and durvalumab in the MYSTIC trial had a median overall survival of 11.9 months but had a higher rate of grade 3 or higher adverse events compared to the durvalumab monotherapy group. Patients in the NEPTUNE trial who underwent durvalumab therapy in combination with tremelimumab yielded a higher 12-month PFS when compared to patients treated with chemotherapy only. Of the patients treated with durvalumab and tremelimumab, 26.1% were alive at 24 months compared to 13.6% in the patients treated with chemotherapy.

Avelumab

Avelumab has been investigated in several clinical trials that include renal cell carcinoma (RCC) and urothelial carcinoma (UC). Merkel cell carcinoma (MCC), a malignant skin neoplasm, has also been investigated in its response to avelumab therapy. In a study by Motzer et al. [8] in 2019, 886 patients with RCC were given avelumab plus axitinib. The PFS of these patients were compared to groups of patients treated with sunitinib, a tyrosine kinase inhibitor, which has been thought to be the standard care of RCC. The results of this study revealed that the PFS of the patients treated with avelumab plus axitinib had a significantly longer PFS compared to the group treated with sunitinib, with PFS values of 13.8% and 8.4%, respectively. A much smaller study published in 2023 involving combination therapy with avelumab and another tyrosine kinase inhibitor, cabozantinib, revealed only preliminary clinical activity. Patient response was measured by the rate of tumor regression compared to baseline. This study involved only 12 patients with renal carcinoma, with one patient showing a complete response and five patients showing a partial response [9].

Avelumab has shown efficacy in the treatment of MCC as evidenced by the JAVELIN Merkel trial. A significant objective response rate (ORR) was observed in groups of previously treated patients and those who underwent avelumab as first-line treatment with values of 33% and 40%, respectively [10]. Studies done in Israel and France also revealed similar outcomes. Within these studies, median overall survival (OS) ranged from 12.6 to 15.9 months and median progression-free survival (PFS) ranged from 2.7 to 3.6 months [11,12]. A study by Ferini et al. [13] in 2023 with eight patients showed promising results of combination therapy involving radiation with avelumab. Ferini et al. [13] reported an ORR of 75% with a one-year survival rate of 85.7% and a two-year survival rate of 64.3%. The results of all these trials come together to show that avelumab, with further research, may become more prominent in the treatment of MCC. Avelumab has been analyzed in the treatment of UC, a neoplasm more common in older individuals who smoke. The JAVELIN Bladder 100 trial revealed a significantly longer PFS in patients undergoing avelumab treatment than those who only had supportive care (5.5 months versus 2.1 months) [14].

Atezolizumab

Lung cancer, correlated with high rates of mortality, has been one of the primary targets for utilizing atezolizumab as an option for oncological intervention. It has largely been compared to the efficacy of docetaxel, a microtubule inhibitor, in the treatment of NSCLC. Major studies reveal that atezolizumab significantly prolongs overall survival in patients compared to docetaxel [15]. Atezolizumab was approved by the US Food and Drug Administration (FDA) to treat patients diagnosed with stage 2-3A NSCLC showing PD-1 expression [16]. Patients with stage 2 NSCLC have cancer that has grown significantly from stage 1 but has not metastasized, while patients with stage 3A have a tumor that is between 3 and 5 cm and has spread to nearby lymph nodes. Atezolizumab has also been implemented in the treatment of small cell lung cancer (SCLC), a much more aggressive form of lung cancer. A study was done that involved the combination therapy of atezolizumab plus carboplatin, a platinum-based alkylating agent, as well as etoposide, an inhibitor of DNA topoisomerase II. The combined therapy demonstrated favorable efficacy in 16 patients with extensive SCLC, supported by a 75% ORR PFS of 5.3 months [17]. The efficacy of atezolizumab has been studied for other neoplasms including urothelial carcinoma. A phase II clinical trial done in 2023 resulted in an ORR of 40.9% with a PFS of 6.9 months for patients with urothelial carcinoma who underwent treatment with atezolizumab and the platinum-based alkylating agent cisplatin [18]. In a retrospective study involving 32 patients, Hussain et al. [19] reported a median PFS of about six months with OS of 10 months. The results of these studies along with others are summarized in Table 3.

**Table 3:** Results of studies investigating the efficacy of atezolizumab for patients with urothelial carcinoma PFS: progression-free survival, ORR: overall response rate, OS: overall survival

Study	Sample size (number)	Median PFS (months)	ORR (%)	Median OS (months)
de Velasco et al. [18]	66	6.9	40.9	6.9
Hussain et al. [19]	32	6	Not reported	10.2
Gritskevich et al. [20]	22	5.2	72.7	18.5
Duran et al. [21]	109	3.7	23.8	8.5
Dominguez Santana et al. [22]	33	5	Not reported	15
Sotelo et al. [23]	127	2.1	20.3	9.2

Atezolizumab has also been investigated in the treatment of breast cancer. The treatment of breast cancer may depend on the expression of receptors such as estrogen, progesterone, and human epidermal growth factor 2 (HER2). Generally, breast cancers that express these receptors may be treated with medications that have been developed to target that specific receptor being expressed by the tumor cells. One trial involved atezolizumab being combined with HER2 monoclonal antibodies (trastuzumab and pertuzumab) and chemotherapy (epirubicin) for treating patients with HER2 early breast cancer. A high response rate (60.3%) was observed in this trial, along with a relatively assuring safety profile [24]. Another trial in 2022 analyzed the efficacy of the atezolizumab agent in treating triple-negative breast cancer (TNBC). TNBC is a very aggressive form of breast cancer because the lack of receptor expression makes it difficult to target with therapy. This study showed that patients with combined atezolizumab and paclitaxel had a favorable PFS and ORR compared to patients who only underwent treatment with paclitaxel [25]. It is important to note that patients in this study who received the combined therapy experienced significantly more side effects compared to those who were treated with paclitaxel monotherapy. The most common side effects included fatigue, nausea, and diarrhea. The most serious side effect reported in the study was febrile neutropenia, which occurred in 11% of patients who were treated with atezolizumab and paclitaxel.

Nivolumab

Nivolumab has shown effectiveness in the treatment of various types of cancers, including squamous cell carcinoma of the head and neck (HNSCC). A study with 46 patients in 2023 revealed that nivolumab was more effective in terms of OS compared to another treatment commonly used: cetuximab, a monoclonal antibody that targets epidermal growth factor [26]. This study also identified several factors that were associated with a positive response to nivolumab. Elevated albumin values, lymphocytosis, neutropenia, and increased neutrophil/lymphocyte ratio values were found to have positive prognostic value in the tumor's response to treatment in this study, although the mechanism has not been clearly understood.

HNSCC is not the only form of squamous cell carcinoma that has been treated with nivolumab. This immunotherapy has also shown efficacy when treating cutaneous squamous cell carcinoma (CSCC), with an ORR of 61.3% in a 32-patient trial by Lang et al. [27]. Another meta-analysis published by Oyenuga et al. [28] in 2022 revealed that patients with esophageal and gastric cancer had improved OS and PFS when undergoing treatment with nivolumab. Patients who had a PD-L1 expression greater than 1% experienced better results with nivolumab. A retrospective and prospective study by Gogate et al. [29] further supported the effectiveness and safety of nivolumab in patients with HNSCC, evidenced by a median OS of 9.2 months and a median PFS of 3.9 months in a sample size of 498. The combination of cabozantinib and nivolumab was tested in a phase I trial for treating genitourinary cancers including UC and RCC. The results showed promising efficacy coupled with an assuring safety profile in patients with these neoplasms [30]. Zhang et al. [31] analyzed the effect of neoadjuvant cabozantinib and nivolumab in the treatment of hepatocellular carcinoma using spatial transcriptomics. The results of this study revealed that there are distinctive and molecular landscapes that can be observed in patients with hepatocellular carcinoma who respond to modern therapy including cancer-associated fibroblasts and increased B-cell expression. An interesting study was published in 2022 investigating the possible prognostic value of liquid biomarkers in patients with melanoma and renal cell carcinoma who underwent treatment with nivolumab. Soluble PD-L1 was found to be higher in patients with progressive or stable disease, indicating that soluble PD-L1 may play a role in refining nivolumab therapy for these cancers [32].

Pembrolizumab

When combined with paclitaxel and carboplatin, pembrolizumab increased PFS in patients with advanced endometrial cancer [33]. This double-blind randomized study included 816 patients and yielded a median PFS of 13.1 months in patients undergoing combination therapy who have endometrial cancer associated with a mismatch repair genetic defect compared to the placebo group. In a study by Strosberg et al. [34], this agent was investigated for the treatment of neuroendocrine tumors in 107 patients. The different types of neuroendocrine cancers included the lung, appendix, small intestine, colon, rectum, and pancreas. The results of this trial indicated limited antitumor activity of pembrolizumab, with only four patients showing a partial response and zero patients showing a complete response. Further research with larger sampling will be needed to analyze whether pembrolizumab may be used in combination therapy to treat neuroendocrine tumors. Few studies have been done to investigate the efficacy of pembrolizumab in the treatment of HNSCC. A summary of the findings is displayed in Table 4.

**Table 4:** Results of studies investigating the use of pembrolizumab for treating HNSCC PFS: progression-free survival, OS: overall survival

Study	Year	Number of patients treated with pembrolizumab	Combination therapy	Median PFS (months)	Median OS (months)
Okada et al. [35]	2023	167	None	5.1	22.7
Pirruccello et al. [36]	2023	39	None	2.9	10
Price et al. [37]	2023	48	PDS0101 (HPV16 targeted immunotherapy)	10.4	Not measurable due to many patients still currently alive
Rodriguez et al. [38]	2020	25	Vorinostat (histone deacetylase inhibitor)	4.5	12.6

The encouraging antitumor activity seen when pembrolizumab was combined with vorinostat indicates a potentially promising combination for utilization [38]. This may also parallel the findings by Price et al. [37]. Larger populations will be required to dictate whether these combination therapies will provide significant benefits on a large-scale basis. It is difficult to determine if the efficacy of combination therapy is proportional, as the study by Okada et al. [35] involved a much larger sample size without combination therapy and yielded comparable results to those studies that did utilize combination therapy. Pembrolizumab has shown noticeable efficacy in the treatment of lung cancer, particularly if combined with chemotherapy. However, certain factors such as sex, age, smoking history, and PD-L1 expression amount may affect the response of lung carcinoma to pembrolizumab therapy [39,40].

Cemiplimab

Cemiplimab has shown promising efficacy in the treatment of different malignancies including NSCLC and CSCC. A real-life study by Hober et al. [41] in 2021 demonstrated a best ORR of 50.4% in a sample of 245 patients who were diagnosed with CSCC and underwent cemiplimab therapy. This ORR combined with a median PFS of 7.9 months significantly underlines the promising future of cemiplimab as an immunotherapeutic agent. Similarly, cemiplimab has shown noteworthy effectiveness in treating NSCLC as monotherapy and in combination with chemotherapy [42]. This is supported by a study where cemiplimab was combined with platinum-based chemotherapy and yielded clinically significant improvements in OS, PFS, and ORR in patients compared to patients who only underwent chemotherapy alone [43,44]. Due to reassuring trials, cemiplimab, when combined with chemotherapy, has been approved by the FDA for the treatment of NSCLC in patients who are not candidates for radiation therapy or surgery, as well as patients with metastatic CSSC.

Resistance to immunotherapy

Medication resistance is a major challenge when treating patients with cancer. Resistance to treatment can result in failure of therapy and relapse of the neoplasm. Resistance may be acquired and influenced by components such as cell influx and efflux variations, mutations, and cell heterogeneity [45]. There are a variety of mechanisms through which tumor cells can avoid clearance, including modulation of drug efflux, alternation of target sites, oxidation, and reduction [46].

PD-1 therapy is subject to treatment resistance in the same way as other standard chemotherapy. Although the trials of using PD-1 inhibitors to treat NSCLC and melanoma have been significant, a large majority of patients develop resistance to the treatment [47]. A particular allelic variant that may lead to resistance of patients with NSCLC to PD-1 inhibitor therapy is the killer cell immunoglobulin-like receptor 3DS1 (KIR3DS1). The presence of the KIR3DS1 allele has correlated with decreased PFS in patients with NSCLC. Although not completely understood, assays have shown that this resistance may be due to natural killer (NK) cell dysfunction resulting in their inability to properly express PD-1 [48]. Further analysis involving randomized trials will be needed to confirm whether this genetic variant is a predictive marker for a patient's response to PD-1 inhibitor treatment.

TYRO3 is a tyrosine-protein kinase receptor that has also been implicated in tumor resistance to PD-1 inhibition [49]. When activated, TYRO3 induces intracellular signaling that enhances cell survival and division. In a study involving mice, high expression of TYRO3 was associated with resistance to anti-PD-1 therapy [50]. Blocking TYRO3 can lead to ferroptosis of tumor cells and may be a significant mechanism to minimize resistance to anti-PD-1 therapy. This process involves an accumulation of iron that results in the generation of many reactive oxygen species. High amounts of these reactive molecules activate lipid peroxidation, leading to the death of the tumor cell [51]. A study by Shao et al. [52] revealed that TYRO3 expression was found to be increased in colorectal cancer cells, and blocking this receptor improved the efficacy of 5-fluorouracil treatment. The findings in this study further suggest that inhibiting TYRO3 may be another promising method to reduce cancer resistance to PD-1 therapy.

Resistance to anti-PD-1 therapy in cancer can also occur through 2,3-dioxygenase-1 expression, leading to immune tolerance and T-cell anergy [53]. Additionally, loss-of-function mutations involving Janus kinase 1 (JAK1), Janus kinase 2 (JAK2), and beta-2-microglobulin (B2M) can result in cancer resistance to PD-1 blockade. The stimulation of NK cells and CD8 T-cell activity may overcome JAK1/JAK2 knockout resistance. B2M knockout resistance may be overcome by activating NK cells and CD4 cells using interleukin-2 (IL-2) agonist bempegaldesleukin [54]. In 2021, Yuan et al. [55] investigated the significance of the V-domain Ig suppressor of T-cell activation (VISTA) as a promising target for immunotherapy to enhance antitumor response in patients with treatment resistance. VISTA downregulates T-cell response and can be expressed in cancerous cells. VISTA monoclonal antibodies have been combined with other agents including PD-L1 inhibitors, Toll-like receptor (TLR) agonists, and tumor antigen peptides, which have led to tumor regression and long-term survival in mice. These findings suggest the importance of further investigating VISTA as a target for effective immunotherapy.

Adverse events

Cutaneous Manifestations

Despite the success of anti-PD-1/PD-L1 therapy being observed in trials, safety profile is an important component that should warrant thorough consideration. There are a variety of adverse events (AEs) reported with the use of these agents. Important AEs to consider are the cutaneous ones, which have impacted more than 50% of patients undergoing PD-1 blockade therapy [56]. In an NSCLC study, Dang et al. [57] reported that the majority of cutaneous AEs included a maculopapular rash, pruritus, and capillary endothelial proliferation. Other reported skin manifestations include morbilliform and psoriasiform rash, bullous pemphigoid eruptions, keratoacanthomas, and lichenoid and spongiotic dermatitis [57,58]. Less common skin effects of anti-PD-1/PD-L1 therapy include alopecia, hypopigmentation, and, in very rare cases, toxic epidermal necrolysis [59]. While most of the cutaneous AEs with this therapy can be tolerable, some patients may refrain from completing full-time treatment.

Immune-Related Adverse Events

Additionally, immune-related adverse events (irAEs) associated with PD-1/PD-L1 inhibitor therapy have been noted in clinical trials. irAEs may involve various organs and systems, including the gastrointestinal tract and nervous system. Gastrointestinal toxicity stands out as a considerable side effect and may present as colitis, perforation, and obstruction. irAE enterocolitis shares similarities with inflammatory bowel disease in terms of endoscopic and histopathological findings [60]. There are no clear diagnostic criteria for irAE gastroenterocolitis, so it is important for pathologists and therapy providers to be aware of this adverse event potentially arising. Although rare, neurological side effects can occur as irAEs in patients treated with PD-1 inhibitors. Reported neurological irAEs include myasthenia gravis, peripheral neuropathy, and encephalitis [61-63]. These adverse events can result in functional limitation, weakness, altered mental status, and other neurological symptoms. Prompt recognition of these symptoms is essential for reducing further neurological complications. Nephrotoxicity, although rare, has been reported as an irAE in patients undergoing this immunotherapy. The most common pathology observed in patients with drug-induced nephrotoxicity is acute tubulointerstitial nephritis, accounting for 72% of cases [64]. Treatment of nephritis typically includes steroid therapy, with most patients responding well and quickly recovering their kidney function. Prompt diagnosis and treatment are critical, as lack of treatment can result in a patient requiring a full transplant with the risk of incomplete recovery [65,66]. Further investigation is needed to improve early diagnosis and develop effective treatment for patients undergoing anti-PD-1/PD-L1 who develop irAEs [67].

Pulmonary Complications

Pulmonary complications such as pneumonitis represent a noteworthy concern for patients receiving anti-PD-1/PD-L1 therapy. Current studies reveal that pneumonitis has occurred in 3.2% of patients undergoing anti-PD-1/PD-L1 therapy [68]. Although it has a very low incidence reported, pneumonitis can escalate and induce respiratory failure in patients with a severe manifestation. Studies show that patients with NSCLC have a higher risk of developing pneumonitis compared to patients with other cancer types. Several other factors can contribute to a patient being more susceptible to developing pneumonitis during treatment. These factors include underlying interstitial lung disease, male sex, and significant smoking history [69]. Treating drug-induced pneumonitis typically involves completely withdrawing or temporarily delaying immunotherapy. Steroids are commonly utilized to reduce inflammation and mitigate any irAEs. Whether the treatment will be continued or modified depends on the severity of pneumonitis and the patient's condition [70].

Future implications

Gaps in Knowledge

Knowledge gaps persist in the discussion of immune checkpoint inhibitor therapy. It is difficult now to predict which patients will respond to a particular PD-1/PD-L1 inhibitor. Advancements in knowledge may provide for future research to be conducted and assess specific biomarkers that can predict and monitor a patient's cellular response to treatment [71]. Research is also still being done to analyze how a tumor's response to anti-PD-1/PD-L1 may correlate with the cancer's PD-L1 expression levels. Although well established in their treatment of certain cancers involving the lung, skin, and genitourinary systems, PD-1/PD-L1 inhibitors have not been shown to provide significant benefits for other cancers, one of them being prostate cancer [72]. Given this neoplasm's high prevalence and mortality rate, future research on immune checkpoint inhibitors should be done to assess their efficacy in treating prostate cancer.

Comparing to the Standard

It is debatable now to decide whether immune checkpoint inhibitors will overcome the utilization of standard chemotherapy to treat cancers. Several considerations must be made such as safety profile, cost-effectiveness, and, most importantly, efficacy. A topic often mentioned in literature is the minimal amount of side effects that come with anti-PD-1/PD-L1 therapy compared to standard chemotherapy. This is an important component as many patients may refuse to undergo treatment for their cancer due to the fear of experiencing debilitating effects such as severe weight loss, diarrhea, vomiting, and hair loss. There are standard regimens in place for specific tumors that have an empirical advantage over anti-PD-1/PD-L1 inhibitors. Even if the side effects of these standard medications may be more prominent, there must be a discussion about the benefit/risk ratio. Based on their efficacy and relatively low incidence of major adverse effects, these agents provide far more benefit compared to risk. A malignant tumor has the potential to quickly evade nearby structures and put a patient in imminent danger of non-treatable, terminal disease. These patients may benefit more from the well-known chemotherapy agents that have been largely studied, regardless of the higher risk of adverse effects.

## Conclusions

Advancements in technology and medical research will be key in the fight against neoplastic disease. Based on the several systematic reviews and results of several clinical trials revealing improved PFS and OS, immune checkpoint inhibitors have a very high potential for being further investigated and to be established as first-line combination therapy or monotherapy to treat cancers. Further research with larger-sized samples will be needed to analyze whether the efficacy of these agents can be consistent and reliable in treating tumors. Successful utilization of these agents in the future will emphasize the importance of immunotherapeutic agents for treating a variety of conditions, with malignancy being the most critical of them.
